# Restoring After Central Nervous System Injuries: Neural Mechanisms and Translational Applications of Motor Recovery

**DOI:** 10.1007/s12264-022-00959-x

**Published:** 2022-11-04

**Authors:** Zhengrun Gao, Zhen Pang, Yiming Chen, Gaowei Lei, Shuai Zhu, Guotao Li, Yundong Shen, Wendong Xu

**Affiliations:** 1 Department of Hand Surgery, Huashan Hospital, Fudan University, Shanghai, 200040 China; 2 The National Clinical Research Center for Aging and Medicine, Fudan University, Shanghai, 200040 China; 3 Department of Hand and Upper Extremity Surgery, Jing’an District Central Hospital, Fudan University, Shanghai, 200040 China; 4 Institutes of Brain Science, Fudan University, Shanghai, 200040 China; 5 State Key Laboratory of Medical Neurobiology and MOE Frontiers Center for Brain Science, Fudan University, Shanghai, 200040 China; 7 Institute of Automation, State Key Laboratory of Management and Control for Complex Systems, Chinese Academy of Sciences, Beijing, 100190 China; 8 Co-innovation Center of Neuroregeneration, Nantong University, Nantong, 226000 China; 9 Research Unit of Synergistic Reconstruction of Upper and Lower Limbs after Brain Injury, Chinese Academy of Medical Sciences, Shanghai, 200400 China

**Keywords:** Stroke, Traumatic brain injury, Spinal cord injury, Brain–computer interface system, Neuroplasticity, Ipsilateral motor control, Axon regrowth

## Abstract

Central nervous system (CNS) injuries, including stroke, traumatic brain injury, and spinal cord injury, are leading causes of long-term disability. It is estimated that more than half of the survivors of severe unilateral injury are unable to use the denervated limb. Previous studies have focused on neuroprotective interventions in the affected hemisphere to limit brain lesions and neurorepair measures to promote recovery. However, the ability to increase plasticity in the injured brain is restricted and difficult to improve. Therefore, over several decades, researchers have been prompted to enhance the compensation by the unaffected hemisphere. Animal experiments have revealed that regrowth of ipsilateral descending fibers from the unaffected hemisphere to denervated motor neurons plays a significant role in the restoration of motor function. In addition, several clinical treatments have been designed to restore ipsilateral motor control, including brain stimulation, nerve transfer surgery, and brain–computer interface systems. Here, we comprehensively review the neural mechanisms as well as translational applications of ipsilateral motor control upon rehabilitation after CNS injuries.

## Introduction

In adult mammals, most corticospinal (CS) neurons (CSNs) in the cerebral cortex project directly to the opposite side of the spinal cord and control contralateral limb movements [1–3]. Therefore, the extremities on the side contralateral to the lesion often display motor deficits following injuries to the cortical descending pathways, such as stroke or traumatic brain injury [3–5]. Among central nervous system (CNS) injuries, stroke is the main worldwide cause of death and disability [6–10]. During the acute phase of injury, the majority of patients experience some degree of spontaneous recovery, thereby the impaired function is partially restored with time [11–13]. After entering the chronic phase of recovery (> 6 months), it is estimated that 55%–75% of stroke survivors exhibit permanent functional impairment of the paralyzed extremities [14, 15].

In recent decades, many efforts have been devoted to rescuing abnormal descending connections in the acute phase; for example, pharmacological interventions, stem cells, behavioral therapies, and non-invasive brain stimulation (NIBS) [11, 16–23]. Although such early interventions can improve parts of motor function, most patients still have difficulty in flexibly using the affected arm and/or hand for completing skilled movements, such as grasping and manipulating objects [24]. Notably, skilled movements of the upper extremities serve as the main predictor of whether a patient will resume a usual professional and personal life after brain injury [11, 24]. Recent studies have revealed that the unaffected hemisphere can compensate for the function of the damaged hemisphere and control the movements of the ipsilateral, paralyzed limb [19, 25–30]. The ipsilateral CS tract (CST) from the unaffected hemisphere to the paralyzed hand is one of the most common routes through which skilled motor function is restored following CNS injuries [30–33]. However, the compensatory ability to regain ipsilateral motor control gradually diminishes with age and is largely determined by the location and extent of the lesion [34–36]. Emerging technologies have focused on using the unaffected hemisphere to control the paralyzed upper extremity, such as novel NIBS strategies, brain–computer interface (BCI)-based rehabilitation systems, and a unique nerve transfer surgery named contralateral cervical seventh nerve transfer (CC7), which can effectively enhance skilled motor performance [27, 37–42].

In this review, we first describe the various effects of CNS injury on the cortical descending pathway and summarize the neural mechanisms of plasticity-dependent motor recovery. We next introduce interventions aimed at protecting or repairing the damaged brain in the early stage and highlight the limitations of their clinical application. Then, we shift our focus from the ipsilesional hemisphere to the contralesional hemisphere and summarize the research advances related to using the unaffected hemisphere to control the paralyzed upper extremity in both animal research and clinical application. Furthermore, we propose a sensorimotor integration-based treatment concept, the individualized, combined application of multiple methods to manipulate sensory input and motor output, which may contribute to driving neuroplasticity and accelerate the recovery of skilled motor function of the paralyzed hand.

## CNS Injury and Plasticity-Dependent Spontaneous Recovery

CNS injuries associated with motor dysfunction results from the cortical denervation due to the destruction of contralesional CST axons [3, 5], also known as the pyramidal tract. The cortical neurons that constitute the CST are also known as upper motor neurons, and postsynaptic neurons of CST in the spinal cord are referred to as lower motor neurons, which are connected to skeletal muscle through neuromuscular junctions and control muscle contraction [1–3, 43]. Anatomically, most CST axons cross the midline in medulla oblongata, forming the pyramidal decussation. During typical development, the CST axons of motor cortices initially project bilaterally to the spinal cord. Continued development is characterized by the progressive weakening of uncrossed CST axons and the strengthening of crossed CST axons through synaptic competition driven by cortical activity [44–46]. Consequently, the uncrossed CST axons target motor neurons innervating the proximal and axial muscles, while the crossed CST axons target motor neurons in the distal muscles and are mainly involved in skilled movements. Most of the CST axons originate from pyramidal neurons located in the deep cortical layers (layers V and VI) of the primary motor and sensory cortex (M1 and S1, respectively). In mammalian species, the terminal distribution of the CST shows marked similarities, but the cortical-motor connections differ among species. In felines and rodents, motor commands conveyed by the CST are eventually transmitted to forelimb motoneurons relayed *via* segmental interneurons and propriospinal neurons [47]. In contrast, direct cortical-motor connections in the distal muscles are a specific feature of primates, thus enabling more advanced hand function and manual dexterity [3, 48–50].

Depending on their location, CNS injuries are divided into upper and lower motor neuron lesions [51–54]. An upper motor neuron lesion refers to an injury/lesion that occurs between the brain and the spinal cord, i.e., proximal to the ventral horn, including cortical damage, internal capsular infarction, brainstem injury, and spinal cord injury (SCI). An injury that occurs in the ventral horn of the spinal cord is considered to be a lower motor neuron lesion. In cortical damage caused by traumatic brain injury and stroke, the CSNs are greatly affected and exhibit degeneration or death, resulting in termination of the transmission of cortical commands [34]. In addition, internal capsular infarction and brainstem injury are primarily found in subcortical regions, known to be vital structures containing CS fibers carrying motor commands from some cortices to lower motor neurons. There are two main categories of SCI: complete and incomplete. Incomplete SCI is commonly caused by compression or damage to the spinal cord, which decreases motor signal transmission. However, complete SCI is the most severe and results from serious trauma to the spinal cord, bilaterally eliminating signal transmission between the spinal cord below the injury site and the brain. Therefore, these types of CNS injury disrupt CS fibers, causing motor dysfunction and paralysis of the limb.

**Fig. 1:**
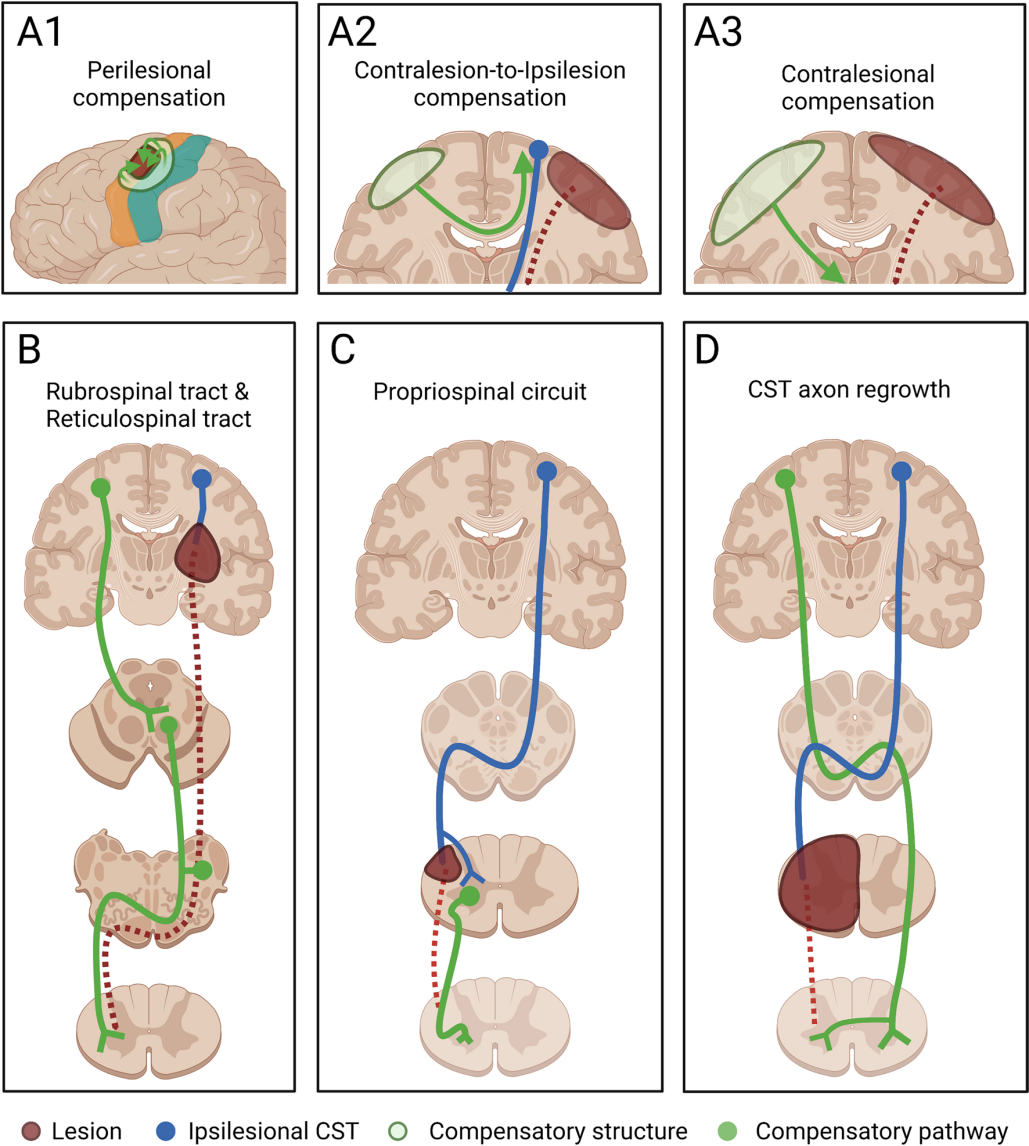
Compensatory pathways during the acute stage after different CNS injuries. **A1** After a local motor cortical lesion, significant reorganization occurs in the perilesional areas to compensate for the injured region. **A2** The contralesional cortex controls the ipsilesional cortex by functional cortico-cortical connections to compensate for the lesion in a moderate cortical lesion. **A3** A severe cortical lesion affects interhemispheric inhibition, while the cortex on the injured side completely loses its function, and the contralesional cortex directly controls the paretic side. **B** The cortico-rubro-spinal pathway is enhanced after damage to the internal capsule or brainstem pyramid. The reticulospinal tracts can be another compensatory pathway after pyramidal lesions. **C** After a DLF lesion**,** the spared propriospinal tract connecting different segments of the spinal cord plays an important role in functional recovery. **D** CST axons from the ipsilesional cortex sprout and cross below the lesion after hemisection of the spinal cord.

In general, CNS injury often results in a permanent loss of motor function, causing mature neurons to typically fail to regenerate. Nevertheless, an accelerating amount of research has revealed that the brain exhibits substantial neuroplasticity during the acute stage after damage, and this can compensate for the damage through reorganization and the creation of new connections among undamaged neurons [55–57]. Injuries occurring in different regions, such as the cerebral cortex, internal capsule, brainstem, and spinal cord, with different lesion extent are compensated for by different alternative pathways [58, 59]. In the event of motor cortical lesions, the perilesional area compensates for the functional loss [55, 60, 61] (Fig. 1A1). In contrast, the contralesional hemisphere exhibits a pronounced compensatory effect for lesions involving sensorimotor cortical areas [25, 59, 62] (Fig. 1A2, A3). The more serous the injury, the greater the activation of the ipsilateral hemisphere that is used during paretic limb movement [24, 63, 64]. Sprouting of the cortico-rubro-spinal pathway contributes to recovery in internal capsule injury [65–67] (Fig. 1B). Likewise, the rubrospinal and reticulospinal tracts play an important part in functional recovery from pyramidal lesions [66, 68, 69]. After a dorsolateral funiculus (DLF) lesion at the C4/C5 segments, an indirect pathway composed of the propriospinal tract contributes to recovery [70] (Fig. 1C). In the event of the hemisection of the lower cervical cord, the CST axons from the ipsilateral cortex descend to the contralesional DLF, and sprout across the midline below the lesion site (Fig. 1D).

Notably, spontaneous recovery is generally incomplete. The degree of spontaneous recovery varies among individuals, and spontaneous recovery usually plateaus 6 months after CNS injury [11, 24]. The majority of patients still have difficulty in flexibly using the affected arm and/or hand for performing motor skills, such as grasping and manipulating objects. Therefore, in recent decades, various restorative therapies have been developed or are under development and aim to potentiate the remaining function of the damaged brain or enhance the compensatory capacity of the undamaged brain by fostering neuroplastic changes.

## Neuroprotection, Chemical Neuromodulation, and Neurorepair Strategies in the Damaged CNS

In patients, neuroprotective agents or endovascular therapy should be administered strictly within the neuroprotection time window, which is 4–6 h after CNS injury [71, 72]. Clinical treatments administered beyond this brief window have limited neuroprotective effects [73, 74]. In the damaged brain, the neuroinflammatory cascade amplifies the primary injury [75, 76]. These secondary biochemical changes lead to tissue damage with associated cell death [77]. The time over these effects occur has been assumed to be hours–weeks, allowing a potential window for interventions.

Numerous experimental studies have suggested that excitation (glutamate and excitotoxicity), radicals (oxidative stress and free radicals), and cellular suicide (activation of apoptotic-like pathways) are the main reasons for the continuous cell death after CNS injuries [78–80]. Recent animal studies have overwhelmingly established that neuroprotective interventions can relieve secondary tissue damage and improve motor function after stroke [16, 81, 82]. These interventions include the pharmacological blockade of neurotransmitter receptors to inhibit cell death pathways [83] and inducing hypothermia [84] or hyperoxygenation [85, 86]. Besides, these restorative processes mainly depend on the severity of CNS injury and are appropriate for treating mild to moderate injuries but are largely ineffective in severe injuries. However, a number of clinical trials have shown that drugs modulating one or more of these mechanisms do not significantly improve the outcome for stroke patients [16].

In recent decades, it has gradually been recognized that neurovascular units (NVUs) play a major part in neuroprotection. The NVU is defined as a complex functional and anatomical structure including neurons, astrocytes, endothelial cells of the blood–brain barrier (BBB), myocytes, pericytes, and extracellular matrix components [87]. The modulation of interactions among different components of the NVU is essential for rescuing the damaged brain at an early stage. For example, enhancing microglial phagocytosis can reduce the brain damage caused by an excessive inflammatory response in brain tissue, avoiding a large degree of neuronal apoptosis and maintaining a number of neurons as much as possible [88]. In addition to affecting microglia, CNS injury can also continuously induce astrocyte reactivation and regeneration to reduce neurotoxic events and encourage scar tissue formation to avoid further injury [89–91]. Moreover, blood vessels, as multi-branched structures, continuously provide relevant nutritional materials to promote the recovery of injured brain tissue [92]. Furthermore, related studies have also suggested that pericytes contribute to maintaining the stability of the NVU and rescuing the normal BBB structure [93, 94]. Overall, neuroprotection in the damaged brain can accelerate functional and structural recovery in multiple animal models by stimulating endogenous neurorestorative events, such as neurogenesis, gliogenesis, angiogenesis, anti-inflammation, and synaptic plasticity.

Although many neurons around the lesion die after CNS injury, some survive but become dormant. These neurons may still have the ability to be excited in response to inputs from spared circuits and pharmacological neuromodulation. Neurotransmitter agonists, such as serotonin, dopamine, and norepinephrine temporarily restore locomotion when applied to paralyzed SCI animals [95]. Furthermore, systemic administration of CLP290, an agonist of the neuron-specific K^+^/Cl^−^ co-transporter KCC2, enhances locomotion in paralyzed SCI mice [96]. CLP290 downregulates the excitability of spinal inhibitory interneurons enabling increased activity in propriospinal circuits to ameliorate the locomotor dysfunction. These drug treatments targeting propriospinal interneurons enhance their activity to promote locomotion by facilitating the transmission of motor signals through propriospinal circuits [96], and by raising the excitability of downstream locomotor networks [97]. But so far, these pharmacological neuromodulations have not been tested in patients.

In addition, neurorepair strategies, such as the transplantation of neural stem/progenitor cells (NSPCs), can also promote neuroprotection by repopulating areas of damaged cells and tissues [98, 99]. NSPCs are multipotent progenitors and can form neurospheres to differentiate into a wide range of cell types, such as neurons, glial cells, and precursor cells. NSPCs tend to differentiate into oligodendrocytes when transplanted into host parenchyma, while they are more to differentiate into astrocytes when transplanted into a lesion site [100]. Following transplantation into the damaged brain, they can induce regeneration of the axons of damaged neurons to bridge the break caused by injury and thereby recover the regular function of nerve cells and restore brain area function [99, 101]. NSPCs transplanted into sites of SCI enable the regeneration of CS axons by maintaining CST neurons in an embryonic growth state [102], and the regenerating axons can form synaptic projections to appropriate NSPCs-derived neurons [103]. These grafted neurons, functioning as interneuronal relays, extend abundant axons for long distances to the host spinal neurons below an injury [104, 105], and these neurons can respond to cortical stimulation or sensory stimulation below the lesion *in vivo* [106]. Recent studies have also reported that NSPCs derived from human pluripotent stem cells (hPSCs) can differentiate into distinct spinal cord neurons, enable robust CS regeneration, integrate various intraspinal and supraspinal systems, and improve functional outcomes after injuries [107–109]. Enhancing activity within the transplanted hPSCs-NSPCs or in combination with rehabilitation training can promote the efficiency and efficacy of cell transplantation therapy for SCI [110, 111]. Although animal studies have revealed that NSPC transplantation is a feasible and promising means of compensating for lost functions, there are still some limitations and potential side-effects in their clinical application, including a large-scale bottleneck in stem cell production and potential allogeneic rejection of the cells [112].

In summary, neuroprotection, neuromodulation, and neurorepair can not only effectively rescue the damaged CST but also induce neuroplasticity to restore the motor function of a paralyzed limb. However, these successes are limited to animal studies, and translation of these interventions from animal studies into clinical practice has yield disappointing results. Clinical failure may be due to insufficient efficacy of current treatments and ignorance of the heterogeneity among patient populations [16]. Particularly, it is still difficult to achieve persistent recovery of motor function by repairing the damaged brain when injury-related recovery enters the chronic stage.

## The Cortical Physiology of Ipsilateral Motor Control

Is it possible to use the intact hemisphere to control the paralyzed limb on the same side? In fact, as early as 1874, Brown-Sequard declared that it was quite possible and that one hemisphere was sufficient to act on both sides of the body [113]. Anatomical and physiological studies have established that almost 15% of CST fibers directly project to the ipsilateral spinal cord without crossing the medulla [1–3, 114]. Notably, the location, proportion, and distribution of the uncrossed CST axons differ by species and stages of development. Continued development is characterized by the gradual weakening of uncrossed CST axons and the strengthening of crossed CST axons through synaptic competition, driven by cortical activity [114]. With the exception of the uncrossed CST axons described above, the unilateral motor cortex can control ipsilateral movements by the corpus callosum, as well as neural networks interconnecting brainstem and spinal cords [115, 116]. Consequently, this indicates the potential involvement of the contralesional hemisphere in the restoration of motor function after unilateral CNS injury.

**Fig. 2:**
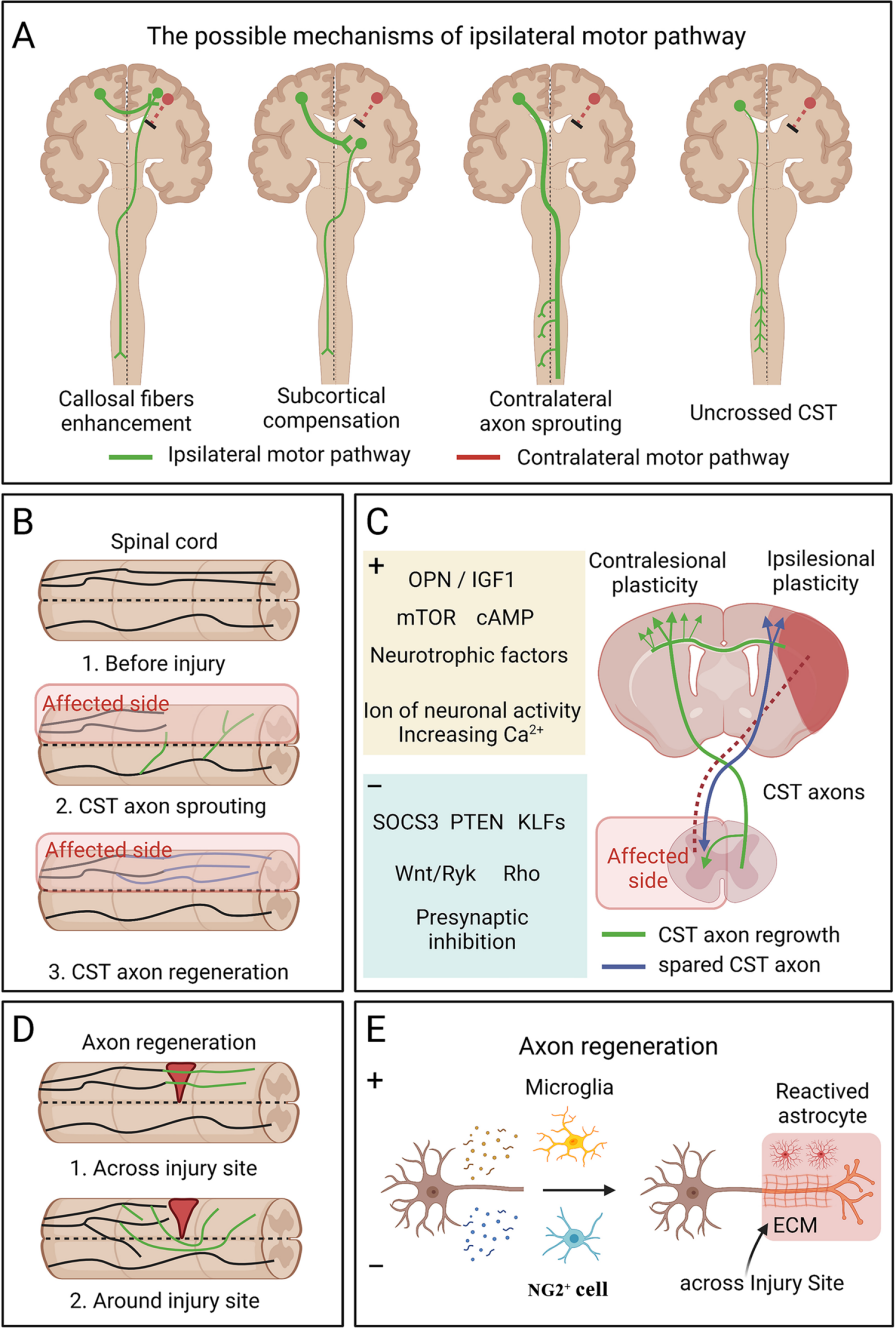
The potential circuit basis and molecular mechanism for implementation of ipsilateral motor control and the cellular mechanism of axon regeneration. **A** Schematic organization of ipsilateral motor pathways. The mechanism of ipsilateral motor control may include the following possibilities: 1. The contralesional cortex controls the remaining CST in the impaired hemisphere by callosal fiber enhancement; 2. Synaptic connections between the contralesional cortex and the ipsilesional subcortical areas are increased; 3. The crossed CST originating from the contralesional cortex sprouts across the midline to the damage-denervated side of the spinal cord; 4. The number and activity of uncrossed CST fibers from the contralesional cortex to the affected spinal cord are increased. **B** Cartoon showing the difference between CST axonal sprouting and regeneration in the spinal cord. Axon growth including axon sprouting and axon regeneration. The sprouting refers to axon regrowth from intact neurons on the unaffected side, and regenerating axons arise from the cut ends of the transected axon of injured neurons. **C** Factors that enhance axon growth are shown in the yellow box, while factors that inhibit axon growth are shown in the blue box. Note that both CST regrowth (green) and spared CST axons (blue) aim to re-innervate the spinal cord of the affected side. **D**, There are two forms of axon regeneration in spinal cord injury, one in which regenerating axons cross through the glial scars, and one in which regenerating axons bypass the injury site. **E** Cellular mechanisms that facilitate the passage of regenerating axons across the astroglial scar. The neonatal microglia secrete fibronectin and their binding proteins to establish a bridge to the extracellular matrix and express various peptidase inhibitors to promote CST regrowth to pass through the injury site. Nerve/glial antigen 2 positive (NG2^+^) cells increase scar formation by secreting pro-inflammatory factors that impede the passage of regenerating axons through glial scars.

It is well known that the juvenile brain has more powerful neural plasticity than the adult brain, thus allowing better functional recovery after CNS injury [117, 118]. Patients who undergo brain injury at a young age often exhibit substantial improvement in motor function [119]. Animal studies have revealed that the ipsilateral motor pathways from the contralesional sensorimotor cortex to the opposite side of the spinal cord play a vital role in motor recovery following a sizable cortical lesion at a young age [30, 118, 120]. The possible mechanisms involved in ipsilateral motor control are as follows: (1) enhancing the neuronal activity of the ipsilateral cortex *via* the corpus callosum and the more substantial contralateral CST [121]; (2) regenerating new synaptic connections of the contralateral CST in subcortical areas such as the brainstem, leading to the reorganization of descending motor pathways [122–124]; (3) promoting axon sprouting of the CST in the contralesional hemisphere across the midline and growing into the denervated area of the spinal cord [2, 33]; and (4) enhancing the efficacy, increasing the regeneration, or decreasing the degeneration of the uncrossed CST axons of the ipsilateral spinal cord [31, 125] (Fig. 2A). Certainly, multiple mechanisms must be involved in the reorganization of ipsilateral motor pathways after injury, and these mechanisms need to be further elucidated.

Unfortunately, such compensatory ability gradually diminishes as the brain matures, mainly because the ipsilateral motor pathway, which perform as compensation for the early stage of development, becomes sparse with maturity [3, 115, 116]. Recent studies have established that the ipsilateral CST is also necessary for behavioral recovery after CNS injury in adult rodents [19, 126], monkeys [127], and humans [128, 129]. However, the regenerative characteristics of the ipsilateral CST are significantly different at different developmental stages. As the ipsilateral CST is not fully pruned after CNS injury in childhood, the axons of CSNs in the uninjured motor cortex project to both halves of the spinal cord to control both limbs [33, 130]. Nevertheless, in adulthood, the decline in cortical neural activity on the injured side promotes axon sprouting, but this compensatory effect cannot flexibly control the impaired limbs by the weakened ipsilateral CST. To achieve ipsilateral motor control after CNS injury, the underlying mechanism is related to the selective activation of CSNs and cortical layer V neurons, promoting neuronal plasticity, and ameliorating injury-induced sensory-motor deficits. Therefore, new therapies are urgently needed to stimulate the compensatory ability within the contralesional cortex to recruit the spared circuit in adult patients with CNS injury.

## Cellular and Molecular Interventions for Promoting Axon Regrowth

By definition, axon growth includes both regeneration and sprouting, with the difference between the two being whether the neuron was damaged in the first place (Fig. 2B) [131, 132]. Regeneration refers to axon growth of damaged neurons, whereas sprouting refers to axon growth of undamaged neurons. It is now widely recognized that both the intracellular and extracellular environments can influence axon growth [133, 134]. Myelin-associated inhibitors (MAIs), such as Nogo and myelin associated glycoprotein, are an important class of neuron-extrinsic mechanisms that inhibit axon growth. Interestingly, inhibition of MAIs alone increases compensatory axon sprouting, but has poor effect on the axon regeneration of injured neurons [135–137]. Notably, research progress in the mechanisms underlying the change of axon growth capacity in the development period contributes to understanding the neuron-intrinsic mechanisms underlying axon regeneration [34, 133]. During development, the axon growth competence of neurons diminishes after synapse-formation is complete and functional neuronal circuits are established. After CNS injury, the cellular processes in the affected neurons are altered, and the neuron transitions from an active, electrically-transmitting state to an electrically-silent, growth-competent state similar to that during development, regaining the intrinsic capacity for axon growth [133, 138]. Next, we summarize the optimal interventions related to axon regrowth from cellular and molecular perspectives.

Recent studies have shown that multiple steps of signal initiation and transduction, including receptor availability [139] and signal propagation [140, 141], or silencing inhibitory pathways [142, 143], can be targeted to enhance axon regeneration of the mature neuron (Fig. 2C). For example, a combination of osteopontin/insulin-like growth factor 1 (IGF1) induces robust sprouting of CST axons and associated behavioral recovery after injuries [144]. In addition, IGF1-dependent axon regeneration is down-regulated by presynaptic neurons [139]. Interestingly, in the case of a lack of growth factors, direct molecular intervention targeting adult neurons promotes robust CST axon sprouting and restores motor function after injury, including enhancers such as WNT/RYK signaling [145], ras homolog gene family member A [146], cyclic adenosine monophosphate [147], and mammalian target of rapamycin [148], and repressors such as phosphatase and tensin homolog [149–151], Kruppel-like transcription factors [152], and suppressor of cytokine signaling 3 [149]. Similarly, some ions involved in neuronal activity play an important part in promoting axon regeneration. An increase of the Ca^2+^ concentration in damaged neurons within minutes after the lesion promotes the initiation of axon regeneration by promoting neuronal growth cone assembly and other stages of axon regeneration [133, 153, 154]. But the continuous Ca^2+^ influx through voltage-gated Ca^2+^ channels after injury suppresses the axon regeneration, and the ablation of *Cacna1c* (Calcium Channel Subunit Alpha1c, encoding the Cav1.2 channel) [155] or *Cacna2d1* (encoding subunits necessary for the Cav2 channel) [156] efficiently promote axon regeneration in dorsal roots ganglion ( DRG) neurons after peripheral axotomy or SCI.

In addition, extensive scar tissue is formed after CNS injury, and newly-formed axons need to reinnervate and redistribute axon terminals through scars (Fig. 2D). The CNS scar is a compartmentalized structure and consist of two major parts: the reactive astrocytes constituting the outer glial scar [157] and the fibrotic scar at the core of the lesion with pericyte derived fibroblasts [158] and inflammatory cells. Recently, different types of loss-of-function study have revealed that scar formation and fibrosis in the CNS involves complex interactions among multiple types of CNS glia and non-neural stromal cells. A partial reduction of Type-A pericytes reduces the fibrosis, enhances the tissue repair and promotes locomotor recovery, whereas a more complete prevention of the scarring produced by Type-A pericytes appears to be detrimental and worsens the outcome [159]. Preventing astrocytic scar formation or specifically ablating chronic astrocyte scars does not lead to axonal regrowth after SCI [160], while pharmacological blocking of the interaction between reactive astrocytes and type I collagen prevents glial scar formation, allowing for a more loosely-arranged glial architecture and promoting axonal regrowth [161]. Besides, astrocytes activated by injury synthesize and secrete various neurotrophic or axon-growth-supporting factors for axon regeneration, including brain-derived neurotrophic factor [162], ciliary neurotrophic factor [163, 164], and chondroitin sulfate proteoglycan 5 (also known as neuroglycan C) [160], which contribute to the composition of the injury microenvironment. Depleting microglia specifically results in an increase in systemic immune cell infiltration and neuronal cell death, disruption of glial scar formation, and worsened behavioral outcomes after SCI [165–167]. Moreover, the latest research has revealed that a subgroup of microglia secrete fibronectin and its binding proteins to establish a bridge to the extracellular matrix and express various peptidase inhibitors to promote scarless healing of spinal cord lesions [168], thus allowing descending axons to pass through the injury site in neonatal mice. Such new insights from cell type-specific loss-of-function studies and next-generation sequencing results have yielded a more complex portrait of the molecular mechanisms governing the glial and neuronal responses to injury, and provide possible combinatorial therapeutic approaches to scar modulation to restore function after severe CNS injury [169].

Notably, robust axon regrowth and the establishment of appropriate neural connectivity does not mean restoration of neural function [170]. Combined rehabilitation training at an adequate time encourages CST regrowth and helps to form, select, and stabilize new functional neural circuitry [19]. Overall, sequentially combining sensory-motor rehabilitation with molecular therapies may act synergistically to enhance cortical plasticity in the intact brain and thereby promote long-term recovery after CNS injury [117]. However, these successes are currently restricted to animal studies and are challenging to translate immediately into clinical applications.

## Clinical Interventions for Enhancing Compensatory Capacity

Current treatments to improve long-term outcomes in CNS injury patients include rehabilitative training, electrical stimulation, and pharmacological interventions [11, 18]. However, these treatments have had only limited success, and it is difficult to achieve the recovery of motor skill at the chronic stage. In recent years, several advanced neuromodulation treatment strategies have been developed to improve motor performance in people with functional impairments [117]. We next summarize the neurotechnologies designed for applying appropriate stimulation to the nervous system to help enhance neuroplasticity in the intact hemisphere, thus facilitating functional recovery of the paralyzed arm/hand.

### Electrical Stimulation to Drives Neuroplasticity for Functional Recovery

Electrical stimulation can be delivered by non-invasive surface electrodes or invasive implanted electrodes to activate or inhibit the CNS. This stimulation can enhance the neural plasticity of specific brain regions, modify local neural circuits in combination with appropriate neurological rehabilitation training, and correct maladaptive neural reorganization caused by neurological injury [11, 18, 171].

**Fig. 3:**
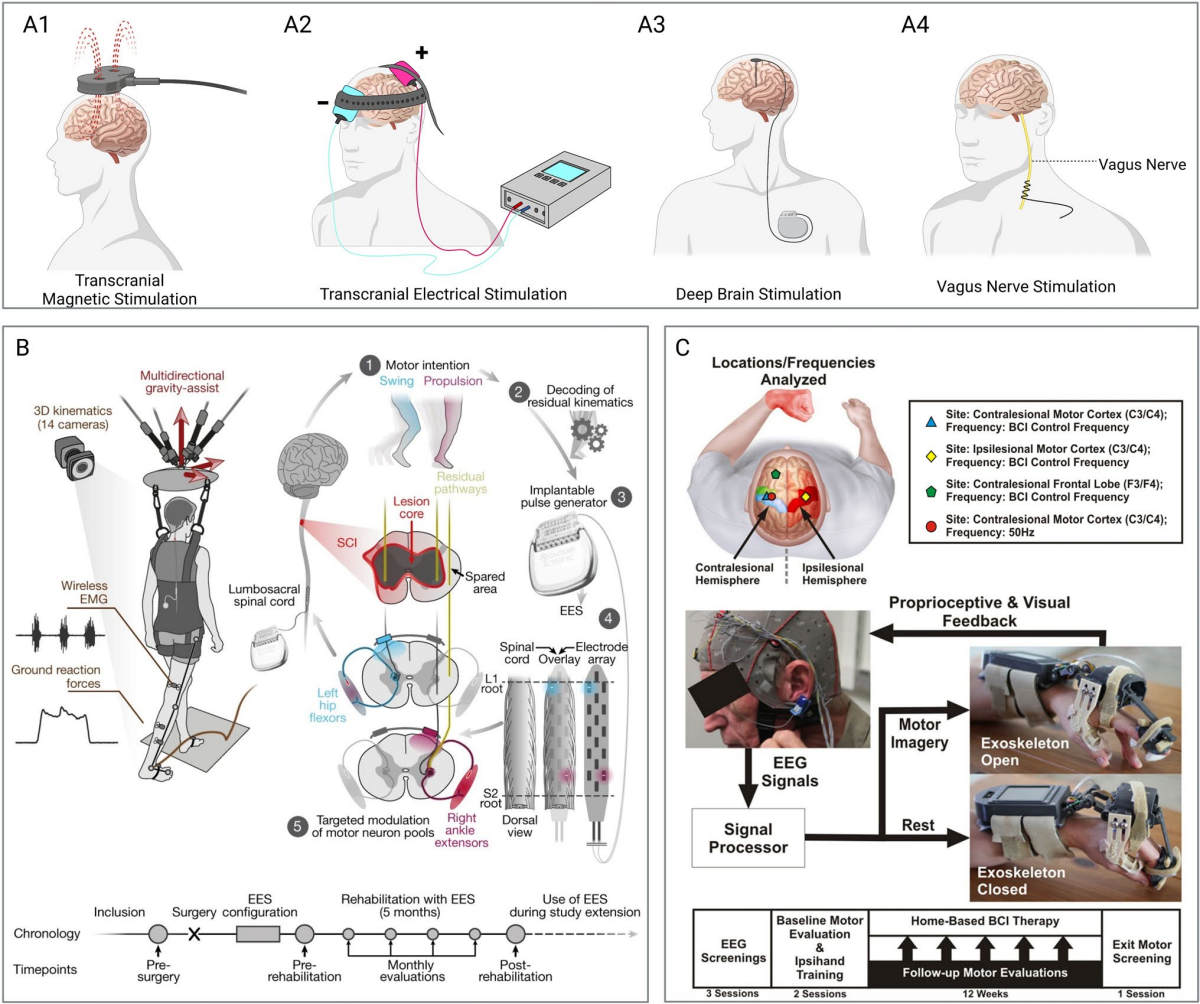
Clinical advances for enhancing neuroplasticity to promote motor recovery. Schematic of brain stimulation systems for patients: **A1** The noninvasive brain stimulation method TMS controls cortical activity *via* magnetic signals. **A2** tES modulate the activation of cortex by low-intensity current. **A3** In invasive brain stimulation systems, such as DBS, electrodes are implanted in a deep brain region and a generator is implanted in the upper chest. **A4** The VNS system requires the implantation of electrodes into the vagus nerve in the left neck. **B** Targeted neurotechnologies for EES during overground walking in patients with SCI [187]. Upper: During training, the wireless communication environment ensures that the EES on the spinal cord can be independently adjusted in real time. An auxiliary device applies multidirectional forces to the trunk against gravity and a real-time processing system records the full-body movements, ground reaction forces, and the electrical activity of leg muscles. A 16-channels electrode paddle array with pulse generator is implanted in the lumbosacral dorsal roots that connect to specific motor neuron pools innervating different leg muscles. EES sequences under voluntary intention induce different lower extremity movements, such as hip flexors and ankle extensors. Lower: The study timeline of this system.** C** The mechanism and study methodology of IpsiHand [37]. Left: The EEG electrode placement strategy of IpsiHand. The recording electrodes are placed in the bilateral motor cortex (blue triangle in the contralesional motor cortex and yellow diamond in the ipsilesional motor cortex), a spatial control electrode (green pentagon) in the contralesional frontal lobe, and a spectral control electrode in the contralesional motor cortex (red dot). Right: The images above show how IpsiHand works. The exoskeleton is attached to a patient’s impaired forearm, palm, and intermediate phalanges of the index and middle finger. A microprocessor in the forearm controls the exoskeleton by an assembly that processes EEG signals. Based on the decoded EEG signals, a linear actuator drives fine hand movements in a 3-finger pinch grip.

Noninvasive brain stimulation (NIBS), including transcranial magnetic stimulation (TMS) and transcranial electrical stimulation, is available to activate or inhibit the CNS by electrodes placed on the epicranium (Fig. 3A1 and A2) [7]. Based on the finding of interhemispheric imbalance, NIBS was first used to suppress the contralesional hemisphere and attenuate transcallosal inhibition to activate the ipsilesional primary motor cortex. However, an increasing number of negative results have appeared, and no additional beneficial effects of the re-activation of the ipsilesional hemisphere have been reported [172]. A plausible explanation is that suppression of the contralesional motor cortex reduces the excitability of the ipsilateral motor pathways that are also important for movement of the paralyzed limb. Several neuroimaging results have demonstrated that the contralesional hemisphere also participates in functional restoration by involving the ipsilateral CST pathway and the cortico-reticulo-propriospinal pathway [40, 65, 173, 174]. We then conclude that NIBS is not the “one size fits all” solution for all patients but that it can be tailored to individuals according to the extent and location of damage, the degree of functional deficiency, or the stage of the recovery process [11]. Moreover, NIBS should not only focus on interventions in the primary motor cortex. In addition, it is notable that the premotor cortex, supplementary motor areas, and the cerebellum, as well as other areas connected to the primary and secondary motor areas and form a complex network, are also involved in the processes of restoration of motor function after brain damage [39, 175]. Therefore, patient-specific characteristics might be considered for determining the best neuromodulation target and more focal stimulation of specific cortical targets. The combination of EEG and TMS has recently offered a new direction named “closed-loop NIBS”, which provides a novel and effective strategy of neuromodulation to restore the impaired motor function [176]. These closed-loop strategy-based therapeutic approaches can successfully restore injured motor function in patients after SCI. Even though various animal experiments and clinical trials have established that NIBS can treat the dysfunction caused by CNS injury to a certain extent, the results are not satisfactory [18]. In future, the underlying concepts of NIBS need to be deeply elucidated, and NIBS-based treatments, such as personalized NIBS, multi-site NIBS, and closed-loop NIBS need to be further developed.

In deep brain stimulation (DBS), the most invasive therapy, electrodes are implanted in brain regions, such as deep nuclei and white matter tracts, and a generator is implanted in the upper chest (Fig. 3A3). The stimulation pattern of DBS is often continuous, and its intensity and frequency can be customized according to individual characteristics. Originally used to treat Parkinson’s disease, DBS can relieve symptoms such tremors, stiffness, and involuntary movements. Recent studies have made a breakthrough in the application of DBS to treat severe traumatic brain injury (STBI) [177, 178]. Direct activation of central thalamic neurons through DBS in patients with STBI tends to normalize cortico-striato-pallido-thalamocortical function. For example, a clinical trial demonstrated that DBS of the bilateral central thalamus alleviated the arousal disorders and increased functional limb control in a 38-years-old patient who remained in a minimally conscious state for 6 years following STBI [179]. Therefore, DBS provides a feasible solution to activate dormant networks or reorganize aberrant or desynchronized connections across brain regions to accelerate functional recovery.

Vagus nerve stimulation (VNS) consists of electrodes connected to the left vagal nerve and a pulse generator implanted under the skin (Fig. 3A4). Previously, VNS was applied in patients with pharmaco-resistant epilepsy, and several studies demonstrated that it triggers neural plasticity to reorganize the motor response area by activating ascending cholinergic pathways [180]. Recently, various animal studies and clinical trials have confirmed that VNS combined with rehabilitative therapies is a promising treatment option for hemiplegic patients [181, 182]. VNS paired with rehabilitative training in adult rats with stroke improves forelimb motor performance in an untrained task, and the treatment effects can persist for several months, even after stopping the treatment [182]. Anatomically, VNS can enhance plasticity in ipsilateral CST regeneration to enhance synaptic connectivity to the muscles of the paralyzed forelimb. Hence, VNS acts synergistically with rehabilitation exercise to restore impaired motor function by enhancing plasticity in the descending motor pathway.

Recent clinical studies of spinal cord epidural electrical stimulation (EES) have demonstrated that delivery of current to the dorsal spinal cord can restore several voluntary movements below the level of a SCI (Fig. 3B) [183–185]. Further investigations have suggested that EES and task-specific training can reconstruct independent stepping in completely paralyzed patients [186–188], revealing the reactivation of previously dormant spared spinal circuits and the enhancement of use-stimulation dependent plasticity [183, 184]. On the other hand, EES can recruit proprioceptive input from afferent dorsal roots, which has been suggested to be central to the ability of EES to engage motor neurons at specific spinal cord segments [187]. Spatiotemporal EES applied to specific dorsal roots allows for selectively stimulation to be timed to coincide with the desired movement [187, 189].

In sum, electrical stimulation therapies are promising; however, based on the individual characteristics of patients evaluated by electrophysiology and imaging, current neuromodulation techniques need to be combined with traditional rehabilitation and necessary pharmacological treatment, ultimately leading to the formation of a complete rehabilitation treatment program for patients with motor disorders after CNS injury.

### BCI-assisted Movement Control

BCI systems record brain signals, analyze them, and translate them into specific commands that are relayed to output equipment that achieves the desired actions [190]. BCI systems record and decode specific signals related to motion execution, and provide appropriate feedback to the CNS for enhancing neural plasticity. To date, BCI systems have been used as promising treatments to restore motor function in patients following chronic CNS injury. Studies have demonstrated that BCI-controlled electrical stimulators targeted to muscles or peripheral nerves and BCI-driven neuroprostheses can effectively improve motor performance in stroke survivors. But these BCI systems for stroke patients have only focused on residual signals from the perilesional cortex [191, 192]. However, in patients with moderate-to-severe cortical damage, it is difficult to modulate perilesional cortical activity, and new therapies should be directed to focus on the non-lesioned, or ipsilateral, cortical hemisphere.

It is universally acknowledged that unilateral limb movements derive primarily from the cortical hemisphere contralateral to the limb, but the ipsilateral motor cortex is also involved in the control of these movement [116]. This ipsilateral motor activation can be used to decode specific motor intentions [193, 194]. Recently, the FDA approved a BCI system, “IpsiHand”, from the company Neurolutions, as a novel and potentially powerful tool that processes movement-related EEG signals from the contralesional hemisphere to control the exoskeleton of the paralyzed hand, which may lead to functional improvements in patients with chronic stroke (Fig. 3C). This contralesional BCI was designed and configured for stroke rehabilitation in the home environment [37, 38]. Despite much progress, the development of this BCI-driven powered exoskeleton system for stroke patients to restore dexterous movements and perform daily living activities remains an ambitious project. The current design mainly has the following deficiencies: (1) This product cannot separate the signals of both upper extremities so that the contralesional hemisphere cannot spontaneously control both the healthy and the paralyzed hands independently. (2) IpsiHand has a flexible exoskeleton only for the index finger with a single degree of freedom and cannot achieve motion assistance for multiple joints of the hand. (3) IpsiHand does not induce neuroplasticity in the contralesional cortex, and the restored functions cannot be maintained without the assistance of the BCI device. Therefore, compared with traditional rehabilitation, the IpsiHand system-assisted rehabilitation improves the Fugl-Meyer (FM) score by only 0.79 points, far from reaching the minimum difference of clinical significance (FM score 5.25 points).

While various interventions targeting the CNS can effectively increase neuroplasticity, peripheral sensory-motor training may play an important part in remodeling brain function. Neuromuscular electrical stimulation (NMES) provides proprioceptive feedback through the residual sensory afferent pathway to reactivate spared cortical circuits and achieve motor recovery [195, 196]. Recent studies have established that an NMES-based BCI approach may achieve functional recovery in patients with moderate and severe chronic stroke, and such recovery can continue to be maintained 6–12 months after completing treatment [197]. In addition to proprioception, the sense of touch is also essential for motor control. A closed-loop BCI uses residual touch signaling from the patient’s own hand to restore the ability to detect object touch and improve motor functions after SCI [198]. Notably, it is difficult to use a prosthesis or perform any simple task without being able to sense the interacting object. In patients with complete SCI, the brain loses all neural connections below the injury site and disrupts sensory-motor control of the extremities. Recent studies have reported a novel bidirectional BCI that records neural activity from the motor cortex and generates sensations by intracortical microsimulation of the sensory cortex [192, 199]. As a result, bidirectional BCI can enable a tetraplegia patient to substantially restore motor function with a robotic limb [192]. Therefore, we believe that bidirectional closed-loop BCI integrating sensory feedback and motor control is a promising strategy for recovering skilled motor function after CNS injury.

### Crossing Nerve Transfer Surgery to Reconstruct Ipsilaterally-based Sensory-motor Function

**Fig. 4:**
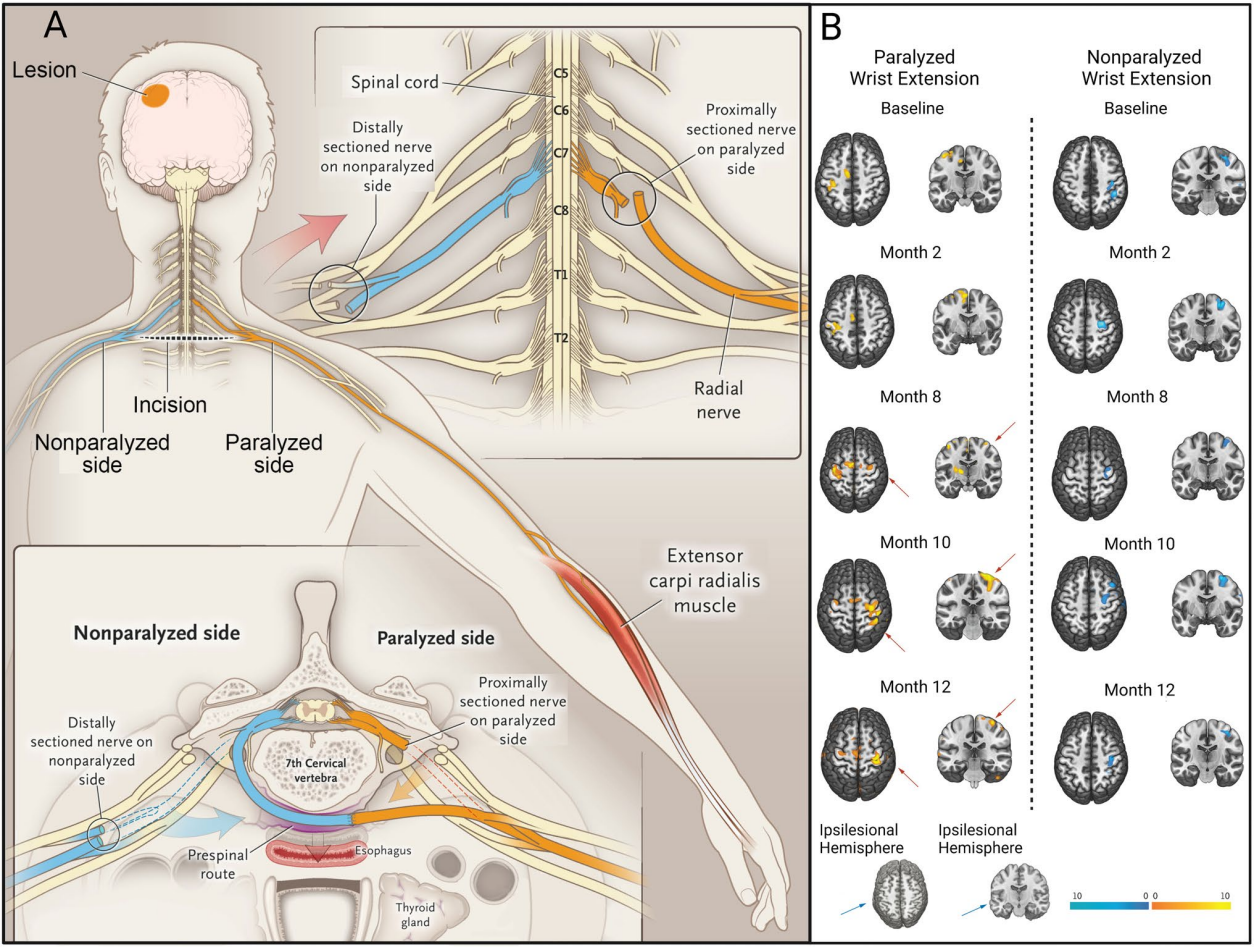
Crossing nerve transfer surgery to improve motor function by enhancing neuroplasticity in the contralesional hemisphere in patients with unilateral arm paralysis [41]. **A** Procedure of contralateral C7 nerve transfer surgery. After harvesting the bilateral C7 nerves in adequate sites, the C7 nerve on the non-paralyzed side (blue) is drawn behind the trachea and esophagus via a pre-spinal route to the paralyzed side (yellow) and coapted directly to the C7 nerve on the paralyzed side. **B** Functional MRI assessment in patients with CC7 surgery. The changes in brain activation on fMRI are evaluated during the 12 months after surgery. Left: Brain activation (yellow) during active extension of the paralyzed wrist. Before surgery, activation was only evident in the ipsilesional hemisphere. At month 8, activation began to appear in both hemispheres. Contralesional activation was enhanced and extended to a larger area than ipsilesional activation at 10 months after surgery, and it was weaker and covered a smaller region at month 12 than at month 10. Right: Brain activation (blue) during active extension of the non-paralyzed wrist. Brain activation associated with movements of the non-paralyzed wrist was stable in the contralesional hemisphere before and after surgery.

The brachial plexus (BP) is a network of five nerves from distinct spinal cord segments (C5, C6, C7, C8, and T1) containing ~ 80,000 nerve fibers and innervating the upper limb [200]. With the widespread use of nerve transfer, we have gained more knowledge of the internal anatomy of individual nerve fibers within the major peripheral nerves. Within the BP, the C7 nerve accounts for ~ 20% and contains both sensory and motor fibers. The unique feature of the C7 nerve is that the its innervation largely overlaps with that of the other four nerves that give rise to the BP. Resection of the C7 nerve usually results in transient weakness and numbness in the upper extremity [201, 202]. Therefore, the healthy side of the C7 nerve in humans can be used as a donor in crossing nerve transfer surgery. Crossing nerve transfer surgery has been widely performed to repair avulsion of the BP by reconnecting the injured nerve ends to the C7 nerve on the healthy side [203, 204]. Recently, we confirmed the possibility of the creative use of crossing nerve transfer as a new peripheral nerve strategy for CNS injury. A crossing nerve transfer surgery, named contralateral cervical seventh nerve transfer (CC7), was applied to achieve significant functional recovery of the paralyzed arm by transferring the C7 nerve from the non-paralyzed side to the paralyzed side in a patient after brain injury (Fig. 4A) [41]. A unique feature of this operation is that the sensory and motor signals of the paralyzed upper extremity communicate with the contralesional hemisphere through the displaced “left-right crossover” nerve. Patients who underwent this surgery showed significant improvements, especially in skilled movements of the paralyzed hand, as measured by Fugl–Meyer assessment; and this recovery led to improved self-care in daily life. In addition, TMS and functional magnetic resonance imaging (fMRI) showed the establishment of physiological connectivity between the contralesional cortex and the paralyzed arm (Fig. 4B). Notably, the functional recovery resulting from CC7 surgery is not limited to function innervated by the C7 nerve itself. Therefore, there are sufficient reasons to believe that CC7 surgery can stimulate neuroplasticity to accelerate motor recovery from CNS injury by modulating peripheral sensorimotor interactions.

## Future Prospects

To date, many emerging neurotechnologies, such as NIBS, BCI, and crossing nerve transfer surgery, have supported the direct modulation of cortical plasticity in the intact brain to improve the fine motor function of the paralyzed limb in adult patients after CNS injury. However, a novel technology is still needed to specifically focus on the coordinated combination of various methodologies to maximize their strengths. Sensory input is quite important for motor function [205]. Sensory signals affect motor functions by transmitting environmental information and intrinsic physiological status as well as by guiding the initiation of the motor system [206, 207]. Nevertheless, the modulation of sensorimotor integration can reactivate dormant plasticity in the adult neocortex. Hence, the next step needs an in-depth exploration of the underlying mechanisms of how sensorimotor integration contributes to skilled hand movements in both normal humans and patients.

**Fig. 5:**
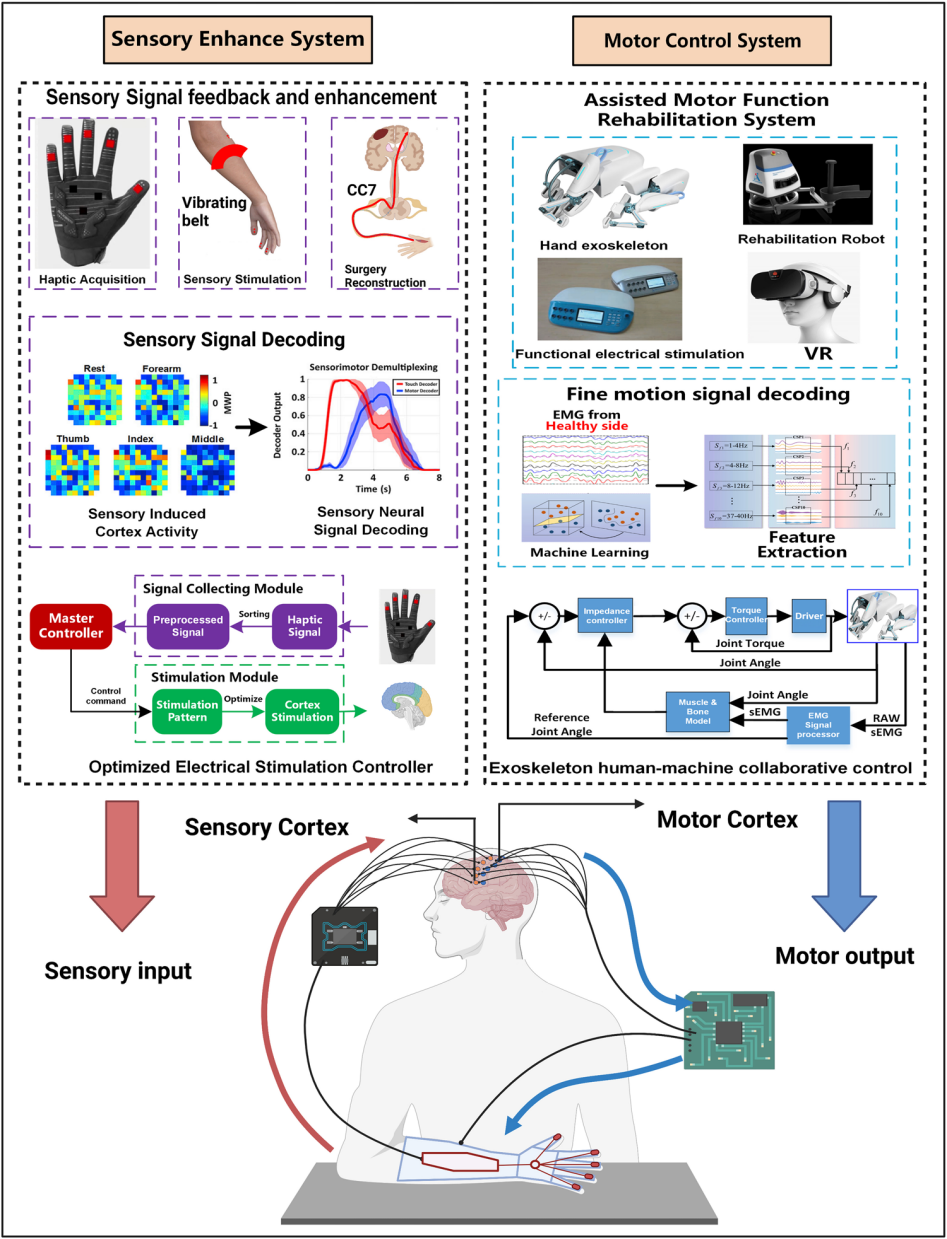
A new rehabilitation strategy that integrates multiple technologies and integrates sensorimotor information to achieve ipsilateral motor control. In patients with unilateral CNS injury, sensory information from the paralyzed limb is increased by using sensors, electrical stimulation, and surgery, and specific sensory information is decoded. The decoded information is then directly transmitted to specific regions of the contralesional hemisphere by electrical stimulation. At the same time, the motor commands from the contralesional cortex are extracted, and the motor information is decoded according to the data set during training, and then the exoskeleton is directly controlled to drive the affected limb and complete the corresponding action.

In this review, we propose that those new therapeutic designs should focus on targeting sensorimotor integration and combining innovative neurotechnologies to drive neuroplasticity in the adult brain to achieve ipsilateral motor control and thus accelerate the recovery of skilled motor performance (Fig. 5). To date, a growing number of amputees are using neuroprosthetics that restore some somatosensation [192, 208–210]. The greatest advantage of crossing nerve transfer is that the sensory inputs from the paralyzed hand can be physiologically transmitted to the ipsilateral (contralesional) brain by avoiding the injured side. Future studies should pay more attention to bidirectional BCI systems, in which sensory enhancement is achieved through peripheral stimulation, surgical reconstruction, or patient-specific cortical stimulation and decoding the signals related to motion intention during motor preparation to control a robotic prosthesis (Fig. 4). We believe that the bidirectional BCI system can be used to maximally enhance intrinsic neuroplasticity while functionally bridging undamaged ipsilateral motor pathways and thus contribute to promoting recovery after brain injury.
